# The Determination of Six Ionophore Coccidiostats in Feed by Liquid Chromatography with Postcolumn Derivatisation and Spectrofotometric/Fluorescence Detection

**DOI:** 10.1155/2013/763402

**Published:** 2013-10-29

**Authors:** Małgorzata Olejnik, Piotr Jedziniak, Teresa Szprengier-Juszkiewicz

**Affiliations:** Department of Pharmacology and Toxicology, National Veterinary Research Institute, Al. Partyzantow 57, 24-100 Pulawy, Poland

## Abstract

The control of levels of anticoccidial feed additives in targeted feeds plays an important role in the assurance of efficiency of animal treatment, prevention of drug resistance, and food safety. The robust and labour-efficient method for the simultaneous determination of six ionophore coccidiostats (lasalocid, maduramicin, monensin, narasin, salinomycin, and semduramicin) in targeted feed has been developed. Properly grinded and homogenized feed sample was spiked with internal standard (monesin methyl ester) and extracted with methanol. The extract was analysed with reversed phase HPLC without any further purification. The separation of the analytes with conventional C18 and core-shell columns was compared. Lasalocid was analysed with fluorescence detection, whereas other ionophores were detected with UV-Vis detector after derivatisation with vanillin in the presence of sulfuric acid. Fortified samples and targeted feeds at authorized levels were used for method validation. Recovery was in the range of 85–110%, depending on the analyte. The within-laboratory reproducibility did not exceed the target value from Horwitz equation. The results of the proficiency tests (*z*-scores in the range of −1.0 to 1.9) confirmed the reliability of the developed protocol.

## 1. Introduction

The intensive modern husbandry practices increase the rate of coccidiosis hence causing large economical losses in poultry production. At the moment, the use of anticoccidial feed additives is thought to be the most effective way of the control of this disease. In the European Union, 11 coccidiostats are authorized as feed additives for poultry and rabbits. The specific documents describe individually for each anticoccidial the target species, applied doses, and conditions of administration, including, when necessary, withdrawal times [1].

**Figure 1:**
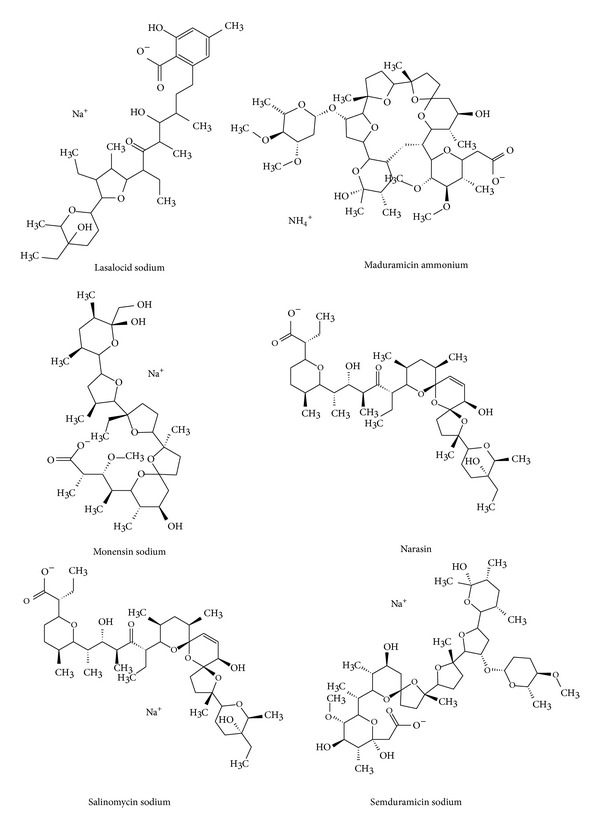
Chemical structures of ionophore coccidiostats.

Among authorized anticoccidial feed additives, ionophore antibiotics (lasalocid, maduramicin, monensin, narasin, salinomycin, and semduramicin) play an essential role (Figure 1). They are used most frequently, due to their low cost and high efficiency. The widespread use of ionophores on the farms can promote the resistant strains of protozoa, resulting in the diminished prophylactic efficiency of coccidiostats; their wide use poses a risk also to human health due to the potential occurrence of residues. The therapeutic index of these coccidiostats is very low, and even target species may be intoxicated when they are exposed to high levels of those compounds. 

In conclusion, to ensure safe and effective use of ionophores, the proper concentrations, precisely as stated in authorization documents, have to be applied. Therefore, the availability of analytical methodology for robust and reliable quantification of ionophores in animal feeding stuffs is essential for both official control and manufacturers' quality control laboratories.

A number of methods for the determination of ionophores in animal feed have been described. Because of the lack of chromophoric groups in their molecules, the derivatisation step is required when spectrophotometric detection is applied [2, 3]. Most of the available methods use postcolumn derivatisation with vanillin [4] or dimethylaminobenzaldehyde, DMAB [5]; such methods are sensitive and robust and require little sample preparation.

Lately, also the mass spectrometric detection has been applied in the determination of coccidiostats in feed [6]. Although MS-based methods can be both used for the reliable determination of single representative of ionophore family [7, 8] and to screen all authorized polyether antibiotics [9], this approach is not always available to routine laboratories because of high cost of purchase and maintenance of mass spectrometer. Also relatively high variation of results is observed due to lack of labelled internal standards.

Some of the methods developed so far, using both postcolumn derivatisation/spectrophotometric detection and mass spectrometric approach, have been validated by interlaboratory comparisons and harmonized as international norms [10–13]. Most of the routine laboratories performing analyses of feed stuffs for ionophore antibiotics follow these official documents. Nevertheless, none of the up-to-date methods enables the determination of all ionophores in single analytical run.

The multianalyte protocols are more practical for the organization of laboratory work in terms of quality assurance and sample throughput. Therefore, we have decided to develop single protocol for the determination of all authorized ionophore coccidiostats based on the postcolumn derivatisation methodology, already successfully applied in the determination of monensin, narasin, and salinomycin [11].

## 2. Material and Methods

### 2.1. Chemicals and Materials

Maduramicin ammonium (MAD), monensin sodium (MON), narasin from *Streptomyces auriofaciens* (NAR), and salinomycin sodium (SAL) were purchased from Sigma (Germany). Lasalocid A sodium (LAS) was obtained from Dr. Ehrenstorfer. Monensin methyl ester (MON-ME) and semduramicin sodium (SMD) were donated by EU-RL in Berlin. 

Methanol, HPLC grade, was purchased from JT Baker (Germany). Methanol p.a., potassium hydroxide, and sulphuric acid (98%) were obtained from POCh (Poland), vanillin from Merck (Germany), and dimethylsulfoxide (DMSO) and potassium dihydrophosphate from Sigma (Germany). Ultrapure water (resistance > 18 mΩ) was obtained from Milli-Q system (Millipore, France). 

### 2.2. Standard Solutions

Stock standard solutions (1 mg/mL) were prepared by weighing 25.0 mg of each reference standard and dissolving in 25 mL methanol. These solutions were kept in the temperature below −18°C for 12 months. Mixed working standard solution (20 *μ*g/mL MAD and 100 *μ*g/mL of remaining ionophores) was stored in 6–10°C up to three months.

### 2.3. Sample Treatment

For the method development and validation, animal feed samples without ionophore coccidiostats were used. The feed was ground with rotor mill ZM200 (Retsch, Germany) and sieved through 0.5 mm sieve. The sample (5 g ± 0.01 g) was weighted into the polypropylene centrifuge tube. The appropriate amount of mixed standard solution was added to the spiked samples, and the sample was let to stand for at least one hour. Methanol was added to the sample so that the total solvent volume was 25 mL (the volume of methanol added with the standard solution was subtracted), and the sample was shaken for 30 minutes at 200 cycles/min (MaxQ 2000 Orbital Shaker, Thermo Scientific, USA). The sample was then centrifuged (3500 rpm, 10 min), and 0.5 mL of supernatant was transferred to a glass tube. Internal standard (monensin methyl ester, 10 *μ*L of 10 *μ*g/mL solution) and 0.5 mL DMSO were added, and the sample was evaporated under the stream of nitrogen (45°C). The extract in DMSO was transferred into a vial and analysed with liquid chromatography.

### 2.4. Instrumental Analysis

The instrumental analysis of coccidiostats was performed using Varian Prostar HPLC system equipped with quaternary pump, autosampler, column oven, postcolumn derivatisation module, and two detectors—fluorescence and UV-Vis, controlled by Galaxie Workstation software. Chromatographic separation of compounds was performed on Kinetex C18 column (150 × 4.6 mm, 2.6 *μ*m, Phenomenex, USA) connected with precolumn (4 × 3 mm, SecurityGuard, Phenomenex, USA). The isocratic elution was applied, with mobile phase consisting of 88% methanol and 12% 0.02 M KH_2_PO_4_, adjusted to pH 7.0 at 0.7 mL/min flow rate. Column oven temperature was controlled at 32°C. After HPLC separation, eluate was transferred through fluorescence detector (excitation and emission wavelength 310 and 420 nm, resp.) for the lasalocid detection. Next, the eluate was passed through the reaction cell (1.4 mL) of the postcolumn derivatisation reactor (Pinnacle PCX, Pickering, USA). The derivatisation was performed with 6% vanillin solution in methanol (0.35 mL/min) in the presence of 4% sulfuric acid in methanol (0.35 mL/min), and the coil was maintained at 110°C. The derivatives of ionophores were then detected at *λ* 520 nm. The injection volume was 50 *μ*L.

### 2.5. Validation

#### 2.5.1. Linearity

Standard calibration curves were prepared by the injection of mixed standard solutions on five concentration levels and plotting recorded peak areas of each analyte versus their concentrations. The equations and regression coefficients of the curves were calculated. Linearity of calibration curves was demonstrated with the *F*-test lack of fit, and the working range was established.

#### 2.5.2. Sensitivity and Selectivity

The selectivity of the method was verified by the analysis of 10 different feed samples (intended for poultry, bovine, and swine). The limit of detection was calculated from the chromatograms of blank samples based on signal-to-noise ratio (S/N value of 3). The limit of quantification (LOQ) was assumed to be at the lowest level of calibration curve; therefore, the repeatability at this level was verified by the analysis of six spiked poultry feed samples.

### 2.6. Recovery and Precision

Blank poultry feed samples were spiked with coccidiostats on three different levels close to the target concentrations specified in the authorisation documents. The spiking levels were 5, 10 and 20 mg/kg for maduramicin and 25, 50 and 100 mg/kg for other ionophores.

For the repeatability study, three series were analysed (six samples for each spiking level). Standard deviation (SD) and coefficient of variation (CV, %) were calculated for each level. The within-laboratory reproducibility was obtained by analysis of two additional series (on all three levels) in the reproducibility conditions (two different occasions, another technician), and overall SD and CV were calculated. 

The coefficient of variation of intralaboratory reproducibility (*n* = 18) was compared with the target deviation according to the equations:

(1) $$\begin{array}{c} \text{Horrat}=\frac{{\text{CV}}_{\text{obt}}}{{\text{CV}}_{\text{tg}}},\\ {\text{CV}}_{\text{tg}}=2^{\left(1-0.5\times \log C\right)}, \end{array}$$

where CV_obt_ is the coefficient of variation of intralaboratory reproducibility from validation data, CV_tg_ is the target coefficient of variation, and *C* is the mass fraction expressed as exponent of 10 [14]. 

The overall mean concentrations obtained in the reproducibility study were used to calculate recovery expressed as percent. 

Additionally, depending on the availability of the samples, the test on the repeatability and within-laboratory reproducibility was performed on target commercial samples.

## 3. Results and Discussion

### 3.1. Method Optimisation

The method presented in this paper is based on the derivatisation approach from ISO norm [11], which proved to be fit for purpose. Still, as the scope of this method is wider in terms of the number of analytes included, the parameters of the detection and separation had to be reoptimised. During the adaptation of detection conditions, two derivatisation agents used commonly in the detection of ionophores were compared: vanillin and DMAB. As expected from bibliographical data [10], DMAB-derivatives gave higher signals. This phenomenon was observed for all ionophores but was most pronounced for the analytes giving high response anyway (especially MON). Therefore, it was difficult to find such conditions of simultaneous detection of all target compounds that MAD and SMD gave acceptable signals and MON did not saturate the detector at the same time. For this reason, vanillin was chosen as the derivatisation reagent, after confirming that it gives acceptable signals for all ionophores.

The next step was the optimization of derivatisation conditions (concentrations and flow rates of reagents, temperature of reaction). Again, the aim of these experiments was to find the conditions optimal for the coccidiostats most difficult to detect. As MAD and SMD are not that easily derivatised as the ionophores included in the ISO method (MON, NAR, and SAL) [11], it was necessary to heat the reaction coil up to 110°C. To prolong the stability of the reagents and avoid the necessity of cooling them, it was decided to prepare both solutions (vanillin and sulfuric acid) in separate bottles.

**Figure 2:**
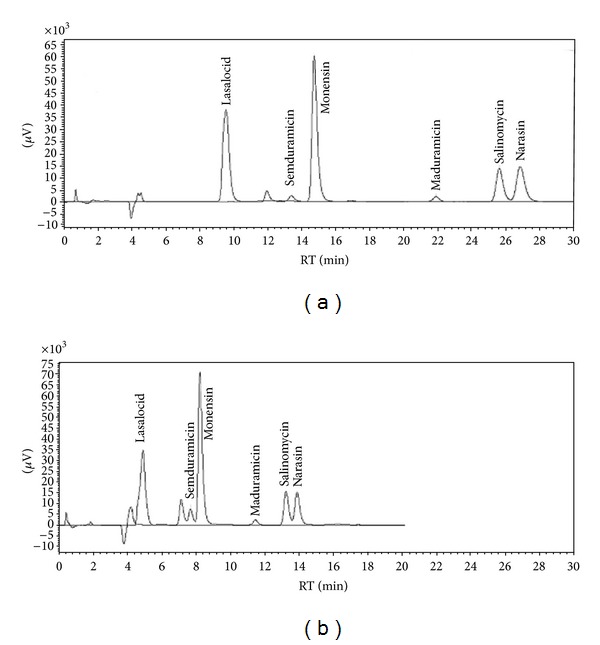
The comparison of the separation of six ionophore coccidiostats with traditional porous column and fused core technology. (a) Chromatogram obtained on Phenomenex Luna C18(2) column (150 × 4.6 mm, 3 *μ*m) with gradient elution of methanol (A) and 0.02 M KH_2_PO_4_ (B) 0–10 min: 88% A, 14–19 min: 90% A, 22–32 min: 88% A; flow rate 0.7 mL/min. (b) Chromatogram obtained on Phenomenex Kinetex C18 column (150 × 4.6 mm, 2.6 *μ*m) with isocratic elution with methanol and 0.02 M KH_2_PO_4_ (88 : 12, *v* : *v*); flow rate 0.7 mL/min.

In the optimization of chromatographic conditions, it was very important to obtain complete separation of semduramicin and monensin. As presented in Figure 2, SMD is eluting between two forms of MON. Potentially, even low levels of monensin could interfere with quantification of SMD, if not separated sufficiently. The acceptable separation was obtained with column Luna C18(2) and very flat gradient of methanol/buffer (details are in Figure 2). With the fused core column (Kinetex C18) shorter analysis time with isocratic elution was obtained. Although it was observed that the resolution was slightly worse in case of Kinetex column, this compromise in chromatographic performance was not significant. Taking into account sample throughput and reagent consumption, this column was used in all the next experiments.

The sensitive detection of vanillin derivatives enables the direct analyses of sample extracts without any purification. In comparison to the ISO norm, only slight modifications of the protocol were implemented. The sample weight and the volume of methanol for extraction were decreased, which reduced the cost of analysis and its impact on the environment. The change of solvent for injection (methanol into DMSO) improved peak shapes and stability of analytes in the extract. 

**Figure 3:**
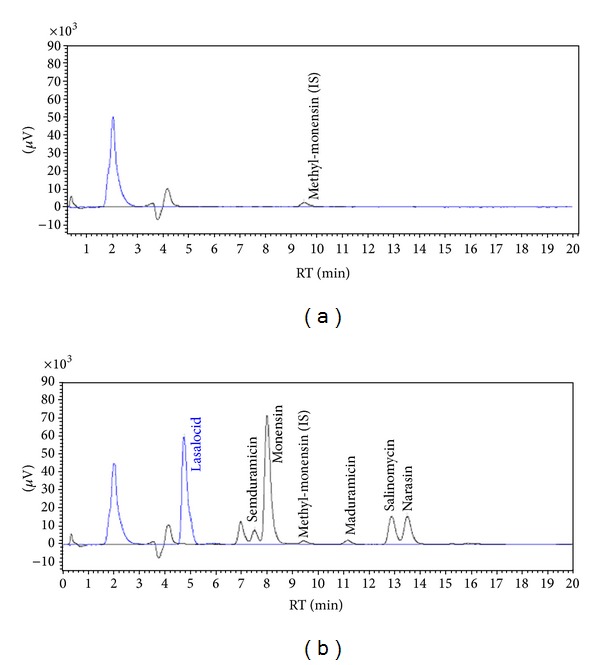
Chromatograms of blank poultry feed sample (a) and feed sample spiked with 50 mg/kg LAS, MON, NAR, SAL, SMD, and 10 mg/kg MAD (b).

As presented in Figure 3, no interferences were observed during analyses of blank samples. The good performance of the method, as well as its labour efficiency, is a consequence of the sensitive and selective detection system. 

### 3.2. Validation and Verification of the Method

During the statistical evaluation of obtained results, it was shown that the precision of the determination of methyl-monensin is much lower than that of other analytes. In consequence, its use as internal standard was not beneficial in terms of quantification. Therefore, it was decided to calculate the results directly from ionophores' peak areas and use methyl monensin only for qualitative control of the analytical process.

The results of validation study are presented in Tables 2 and 3. The linearity range covers concentrations included in authorization documents (Table 1). The analysis of blank samples proved the selectivity of the method and its acceptable sensitivity (Figure 3). 

The obtained values of limit of quantification (1.0–5.0 mg/kg) are slightly higher than the ones from normalized methods [10–13]. A lower limit of quantification is possible to achieve; it was not, however, included in validation study. 

The recovery and precision evaluation was performed using the standard scheme, applied also in the residue control [15, 16]. The criterion for the acceptability of the method was the Horrat value, which should not exceed 1 in reproducibility conditions. In the case of this study, where only inhouse validation was performed, it was expected that the variation would be closer to repeatability target value (around 0.66). As it may be seen from Table 2, the highest obtained Horrat is 0.73. 

In the case of the analyses of commercial target samples, the precision of the protocol is slightly lower. As the sample treatment is really easy and straightforward, the extraction efficiency seems to be the key factor influencing the method performance. Since also these commercial samples results are acceptable, they prove the fitness for purpose of the presented protocol. 

Also the previously published methods using postcolumn derivatisation approach [11–13] give reliable results and are fast and easy to perform. In contrast, the application of other derivatisation protocols makes it impossible to omit the cleanup with solid phase extraction and often significantly impairs both qualitative (limit of detection) and quantitative (precision) performance of the methods [3, 17].

The developed method was successfully verified externally, by the proficiency tests organized by Ducares (The Netherlands). National Veterinary Research Institute has participated in two rounds of the programme concerning the determination of monensin and salinomycin in feeds and obtained *z*-scores from −1.0 to 1.9.

## 4. Conclusions

The authors present an effective method for the determination of polyether ionophores in the feed. Thanks to the modification of chromatographic separation (use of core-shell column and mobile phase at pH 7.0) and derivatisation step (increased temperature of the reactor) in comparison to ISO norm, the developed method allows simultaneous determination of six polyether antibiotics. The results of validation and proficiency test confirm the applicability of the method in the routine feed analysis. 

## Figures and Tables

**Table 1:** The authorization of ionophore coccidiostas in European Union (as for 09/04/2013) [1].

Coccidiostat	Species or category of animal	Content range, mg of active substance/kg of complete feedingstuff
Lasalocid	Turkeys (<12 weeks)	90–125
	Chickens for fattening	75–125
	Chickens reared for laying (<16 weeks)	75–125
	Pheasants, guinea fowl, quails and partridges except laying birds thereof.	75–125
Maduramicin	Chickens for fattening	5-6
	Turkeys (<16 weeks)	5-5
Monensin	Chickens for fattening	100–125
	Chickens reared for laying (<16 weeks)	100–125
	Turkeys (<16 weeks.)	60–100
Narasin	Chickens for fattening	60–70
Salinomycin	Chickens for fattening	50–70
	Chickens reared for laying (<12 weeks)	50–50
Semduramicin	Chickens for fattening	20–25

**Table 2:** The results of in house validation: sensitivity and linearity data.

	LOD, mg/kg	LOQ, mg/kg	Range, mg/kg	Calibration curve	*R*
LAS	0.47	5.0	5–200	*y* = 1700*x* − 254	0.999
MAD	0.65	1.0	1–40	*y* = 376*x* − 11.3	0.999
MON	0.34	5.0	5–200	*y* = 1996*x* − 33.4	0.999
NAR	0.23	5.0	5–200	*y* = 568*x* − 157	0.999
SAL	0.25	5.0	5–200	*y* = 5121*x* − 61.2	0.999
SMD	0.42	5.0	5–200	*y* = 208*x* − 3.484	0.999

**Table 3:** The results of in house validation: recovery and precision of the determination of six ionophore coccidiostats in feed samples.

Coccidiostat	Target concentration, mg/kg	Mean concentration, mg/kg	Recovery, %	RSD_r_, %	RSD_R_, %	Horrat*
LAS	5.0 25 50 100 CFS	5.42 24.3 44.4 85.8 60.9	109 97 89 86 —	6.5 2.0 0.4 1.7 1.7	— 4.0 2.8 3.4 5.9	0.50 0.40 0.32 0.43 0.68

MAD	1.0 5.0 10 20	1.10 4.83 10.5 19.2	110 97 105 96	5.2 2.5 1.7 1.6	— 5.1 5.4 3.4	0.33 0.41 0.47 0.33

MON	5.0 25 50 100	5.00 23.2 49.7 94.3	100 93 99 94	2.8 2.0 1.3 1.0	— 4.4 5.3 2.7	0.18 0.44 0.59 0.34

NAR	5.0 25 50 100 CFS	4.60 23.3 47.7 91.2 47.2	92 93 95 91 —	5.4 2.4 0.4 4.0 3.8	— 4.6 1.3 4.1 6.5	0.42 0.47 0.14 0.51 0.73

SAL	5.0 25 50 100 CFS	4.52 23.9 50.5 91.1 63.3	90 95 101 91 —	3.2 2.3 1.5 4.8 3.6	— 4.3 5.2 5.1 5.2	0.25 0.44 0.58 0.64 0.61

SMD	5.0 25 50 100	4.65 21.3 48.5 89.6	93 85 97 90	6.6 1.3 2.0 1.1	— 5.8 3.6 3.2	0.51 0.58 0.40 0.40

RSD_r_: relative standard deviation of repeatability, RSD_R_: relative standard deviation of in house reproducibility.

CFS: commercial target feed sample.

*Horrat ratio is calculated from repeatability for the lowest spiking level and from in house reproducibility for all other samples.

## References

[1] European Union Register of Feed Additives pursuant to Regulation (EC) No 1831/2003.

[2] Blanchflower WJ, Rice DA, Hamilton JTG (1985). Simultaneous high-performance liquid chromatography: determination of monensin, narasin and salinomycin in feeds using post-column derivatisation. *Analyst*.

[3] Tavčar-Kalcher G, Pavšič-Vrtač K, Vengušt A (2009). Validation of the procedure for the determination of maduramicin in concentrates, premixes, and feeds by liquid chromatography. *Food Additives and Contaminants Part A*.

[4] Coleman MR, Moran JW, Mowrey DH (1997). Liquid chromatographic determination of monensin in premix and animal feeds: collaborative study. *Journal of AOAC International*.

[5] Vincent U, Serano F, de la Huebra MJG, von Holst C (2012). Determination of semduramicin in poultry feed additive, premixture and compound feed by liquid chromatography and UV spectrophotometric detection after post-column derivatisation. *Journal of Pharmaceutical and Biomedical Analysis*.

[6] Vudathala D, Murphy L (2012). Rapid method for the simultaneous determination of six ionophores in feed by liquid chromatography/mass spectrometry. *Journal of AOAC International*.

[7] de la Huebra MJG, Vincent U, von Holst C (2010). Determination of semduramicin in poultry feed at authorized level by liquid chromatography single quadrupole mass spectrometry. *Journal of Pharmaceutical and Biomedical Analysis*.

[8] Dai SY, Herrman TJ (2010). Evaluation of two liquid chromatography/tandem mass spectrometry platforms for quantification of monensin in animal feed and milk. *Rapid Communications in Mass Spectrometry*.

[9] Vincent U, Chedin M, Yasar S, von Holst C (2008). Determination of ionophore coccidiostats in feedingstuffs by liquid chromatography-tandem mass spectrometry. Part I. Application to targeted feed. *Journal of Pharmaceutical and Biomedical Analysis*.

[10] Campbell H, Nayeri G, Costa JM (2006). Determination of monensin, narasin, and salinomycin in mineral premixes, supplements, and animal feeds by liquid chromatography and post-column derivatization: collaborative study. *Journal of AOAC International*.

[11] Animal feeding stuffs Determination of monensin, narasin and salinomycin contents. Liquid chromatographic method using post-column derivatisation.

[12] Animal feeding stuffs Determination of maduramicin-ammonium by reversed-phase HPLC using post-column derivatisation.

[13] Animal feeding stuffs Determination of semduramicin content. Liquid chromatographic method using a “tree” analytical approach.

[14] Thompson M (2000). Recent trends in inter-laboratory precision at ppb and sub-ppb concentrations in relation to fitness for purpose criteria in proficiency testing. *Analyst*.

[15] Thompson M, Ellison SLR, Wood R (2002). Harmonized guidelines for single-laboratory validation of methods of analysis (IUPAC Technical Report). *Pure and Applied Chemistry*.

[16] (2002). Commission Decision 2002/675/EC of 12 August 2002 implementing Council Directive Directive 96/23/EC concerning the performance of analytical methods and the interpretation of results. *Official Journal of the European Communities*.

[17] Dusi G, Gamba V (1999). Liquid chromatography with ultraviolet detection of lasalocid, monensin, salinomycin and narasin in poultry feeds using pre-column derivatization. *Journal of Chromatography A*.

